# Croupus Pneumonia a Specific Infectious Disease

**Published:** 1879-06-20

**Authors:** A. G. Hobbs

**Affiliations:** Indiana


### Southern Medical Record.

EDITORS:

T. S. POWELL, M.D. W. T. GOLDSMITH, M.D. R. C. WORD, M.D.

*R. C. WORD, M.D., Managing Editor.*

☞All Communications and Letters on Business connected with the RECORD must be addressed to the Managing Editor.

VOL. IX. ATLANTA, GA., JUNE 20, 1879. No. 6.

### ORIGINAL AND SELECTED ARTICLES.

### *CROUPUS PNEUMONIA, A SPECIFIC INFECTIOUS DISEASE.*

BY A. G. HOBBS, M. D., OF INDIANA.

Perhaps there is no other disease whose history can show such a variety of medical opinions, and none other is a better example of the great advancement of our science in etiology and pathology than croupus pneumonia. Yet to-day, notwithstanding the advanced views on the pathology of this disease, the treatment has, by no means, kept pace with the pathological advancement.

Improved therapeutics, though, seldom fail to follow quickly in the footsteps of advanced knowledge in this branch.

It is strange that the great Sydenham of the seventeenth century fell so far short of an accurate description of this disease when the ancient Hippocrates described it so nearly in accordance with modern views.

I shall restrict myself in this paper to the discussion of this form of pneumonia and shall occupy the most of it in giving my reasons for believing in its specificicity. It shall be my object to present a resume of the late writings upon this subject, especially those of the Germans, rather than to introduce anything original.

Contrary to the old idea that pneumonia is a local disease with constitutional symptoms, I believe it is now the generally accepted opinion of those who have studied the disease closely that it is a constitutional disease with localized symptoms. The specific morbific element expending itself principally upon the lungs, making those organs the seat of the localized symptoms, just as are Peyer's patches the seat of those symptoms in typhoid fever.

Croupus pneumonia belongs to that class of diseases which are very widely distributed. It regards not degrees of latitude and is not more frequent in one clime than in another. It may be found in the tropics and in the frigid zone as well as in the temperate. This is not the case with other pulmonary diseases, but, on the contrary, catarrh, bronchitis, pleuritis, etc., increase in frequency as we pass from the tropics to the higher latitudes.

The season of the year has a very different effect upon the frequency of pneumonia as compared with catarrh, bronchitis and pluritis. From the London mortality tables which give 5,600 cases pneumonia, 2,300 cases catarrh and 2,300 cases pleuritis, we find the two latter diseases much more uniformly distributed throughout the year than pneumonia (when I use the term "pneumonia" unqualified, the croupus variety is meant.)

More than two-thirds of all the cases that occur during the year are met with during the winter and spring months. Croupus pneumonia occurs in annual cycles just as endemic diseases do generally and a remarkable coincidence is found to exist between its prevalence and that of typhoid fever.

These characteristics, of themselves, do not prove croupus pneumonia to be a specific disease, but they indicate something radically different from the other pulmonary disorders and at least lead us to study the disease more closely.

The communities which live mostly out-of-door lives are less liable to croupus pneumonia than those whose occupations are carried on indoors; this is proved by English statistics as well as the most reliable United States statistics we can find.

The rural districts of England give eight deaths to every 10,000 of the population, while the cities give 20. The meagre statistics upon this subject in the United States give many more cases according to the population in the cities than in the country districts.

Now since all diseases of an infectious nature are found much more abundantly in the thickly populated districts than in the country, this fact alone tends towards the infectious nature of pneumonia. Every one knows that this is not the case with catarrh, bronchitis, etc., but just the reverse is true.

It is an old belief that those who have strong constitutions are especially liable to contract pneumonia, but if we will consult the statistics of our prisons and hospitals we will find that the reverse of this is true. A strong constitution protects against the disease. Convents, prisons and hospitals are often scourged with the disease when the sailor, who is exposed to all the vicissitudes of weather, goes free; to find a single case amongst this class is exceptionable; and it is an acknowledged fact that diseases which are due to specific causes are much more liable to attack those of a weakly constitution. Dietl found only 18 per cent. of previously healthy persons out of 750 cases of pneumonia, and Huss only 16 per cent. out of more than 900 cases.

It is customary for those who still hold to the old belief that pneumonia is a disease of local character to enumerate the exciting causes as follows: Chest contusions, perforating wounds, foreign bodies in the bronchi, a continued breathing of dust, a sudden chilling of the body, etc. Now, the autopsies of all cases on record which were said to have been caused by chest contusions have shown but a localized inflammation of the lung tissues in the vicinity of the contused parts, the lung tissue no more inflamed than the contiguous parts. This cannot properly be called pneumonia; it presents too few of the symptoms of that disease.

Out of 8,760 non-penetrating wounds of the chest taken from the army statistics of the late internecine war but six deaths occurred from pneumonia, a number so small that we cannot attribute the wound as the cause.

Inflammation of the lung tissue brought about by penetrating wounds present but few of the symptoms of pneumonia. The inflammation seldom extends beyond the immediate vicinity of the wound, and there is, I believe, but two cases recorded where the inflammation brought about by this cause extended to the opposite lung, and it is reasonable to suppose that it was but a coincidence in both of these cases instead of a cause.

I believe the whole idea of traumatic pneumonia to be mythical; of course a lung may become inflamed from a traumatic cause, but the inflammation is not pneumonia; it is simply the means that nature always employs to repair a traumatism.

From the meagre statistics that I have been able to compile, stone cutters, wood sawers, and others whose occupations continually expose them to air impregnated with dust and sand are no more liable to this disease than other classes.

The idea that pneumonia is ever caused by a chilling of the body does not seem to me to be tenable, and of 186 cases studied by Ziemsson with reference to this point, he states that it was possible for only ten of these to have been caused by the chilling of the body. Though every practitioner knows that by far the large majority of cases are ushered in with a rigor, yet should we not place the chill under the head of symptomatology than etiology? If a chill be the primary cause of pneumonia, should we not be continually looking for that disease after every sudden chill we meet?

Why should it not then be much more frequent in the intensely malarious than in the non-malarious belts? It has never yet been proven that croupus pneumonia can be produced by any of the usual causes of inflammation. As in typhoid fever there *must* be a special exciting cause.

According to Juergensen during the whole course of croupus pneumonia, there is no relation between the local and the febrile symptoms nor dependence of the one upon the other. This is evident to all who have closely watched the disease at the bed-side. When but one lobe, or even a part of a lobe is involved, we find the severest and most continuous fever, and, on the other hand, both lungs may be extensively complicated, and yet the febrile symptoms be very moderate. How are we to reconcile these facts if we attribute the fever and other constitutional symptoms to the local inflammation in the lungs? Would it not be much more rational to attribute the whole line of symptoms to some specific element centralizing its power principally upon the lungs as we do in typhoid fever where we acknowledge the infection to be centralized upon the agminated glands?

To afford us an additional proof, no affection which arises from a local lesion presents so typical a course in point of time as is the case in croupus pneumonia. All the diseases which we regard as due to some specific cause are characterized by a certain duration in point of days, all the symptoms remaining as long as the virus retains its hold upon the organism, and just as soon as it begins to lose its sway, the recuperative powers of nature step in and assume the reins, and if vitality has not been taxed too severely convalescence is established.

Croupus pneumonia belongs to that class of diseases that are said to terminate by crisis, and, in fatal cases, not by asphyxia as is the case with catarrhal pneumonia and some other pulmonary diseases.

As the fibrinous exudation in the lungs produces an increased resistance in the pulmonary circulation, and consequently an increased effort on the part of the heart, and as the continuous high fever calls for a more abundant interchange of gases, the heart is still farther called on for increased action to supply the deficiency, then we should look for the danger that threatens the heart and expect death from insufficiency of the right ventricle.

Catarrhal pneumonia is but the extension of an inflammation of the bronchi to the pulmonary tissue, or the result of the closure of the inflamed bronchi, and consequent collapse of that portion of the lung supplied by those bronchi. Here there is an exudation of serum only, while in croupus there is an exudation of both serum and fibrin. Interstilial pneumonia is simply an inflammation and hyperæmia of the connective tissue of the lungs.

The catarrhal form of pneumonia presents an entirely different clinical history from the croupus form. In it, *cæteris parabus*, the severity is exactly in proportion to the amount of lung tissue involved; in croupus no such proportion exists. Here death occurs from insufficiency of the respiratory organs; in croupus it occurs at the heart.

As stated before, croupus pneumonia terminates by crisis which, I believe, is a peculiarity of all specific infectious diseases. Diseases of this class have a regular course, a typical temperature, and have generally a definite duration. Under this head we would place the disease in question, together with acute articular rheumatism, cerebro-spinal meningitis, typhoid fever, etc.

Non-specific and non-infectious diseases are fluctuating in their course, irregular in their temperature, and indefinite in their duration. Under this head, we would place catarrhal pneumonia, bronchitis, pleuritis, etc.

Another feature which is peculiar to specific diseases is observed in croupus pneumonia, *i. e.* the peculiar rise in the temperature which occurs in the beginning of defervescence. This is thought to be the partial reabsorption of the *materies morbi*, eliminated into the pulmonary tissues during the course of the disease.

The morbific agent of this disease cannot be isolated, hence we have been obliged to study its etiology in comparison with other affections known to be non-specific and non-infectious; for this reason I have made my comparisons principally with bronchitis, pleuritis, etc., since they have always been regarded as bearing a very close relationship to pneumonia.

These with many other reasons for the specificicity of croupus pneumonia could be given, but my space does not admit of an exhaustive treatise upon the subject, and my object is only to call a few leading facts to the attention of those who reason. I think that any one who will lay aside all preconceived opinions and impartially study this disease, not only from a clinical but from a theoretical standpoint, will come to the same conclusion that I have, *i. e.* that unless quantitative changes in one thing are followed by quantitative changes in another, there can be no causal connection between those things.

Now this is not the case if we regard the local symptoms as the cause of the constitutional, for during the whole course of pneumonia there is no constant relation between the local and febrile symptoms, nor dependence of the one upon the other, hence the constitutional are not the sequence of the local symptoms, but without a doubt the local symptoms are the sequence of the constitutional.

Now, since most diseases which are ushered in with constitutional symptoms are due to some specific cause, it is nothing more than logical, with many other points in its favor, that we should call croupus pneumonia a *specific* disease, and specific diseases are, as a general rule, infectious.

In regard to the treatment, I shall make only a few brief remarks. It is my opinion that those who claim to have aborted pneumonia have not recognized its different forms; of course it is utterly futile to undertake to abort croupus pneumonia if it be of a specific and infectious nature. It may be, and I believe is possible sometimes, to abort catarrhal pneumonia, especially in children, with vigorous treatment begun in its incipiency. Quinine and veratrum, pushed almost to emesis, I am confident will sometimes accomplish this end. Croupus pneumonia passing through all its stages in a very mild form and in an extraordinarily short time may have the appearance of having been "aborted."

Perhaps before proceeding farther I had better give what could be regarded a typical case of croupus pneumonia ending in convalescence. The patient previously having had no warning is generally attacked by a chill, and, in the majority of cases, at night; this is immediately followed with a high fever, a flushed face, a headache, a pain in the side, rapid breathing and a dry hacking cough. If the patient is visited in a few hours, we find him lying upon his back with his head elevated to facilitate breathing; the alæ nasi are moving; the face has an anxious expression and is slightly cyanosed; when he coughs he presses his side with his hand to ease the pain. By examining the pulse we find it ranging from 110 to 130, and the thermometer in the axilla indicates a temperature varying from 102½ to 105, sometimes much higher. During the first 12 or 24 hours the physical signs do not indicate much; there may be a slight vesicular murmur. As the disease advances these signs are gradually intensified, the pulse and the temperature undergo a more or less diurnal change; the sputa turns from a whitish mucus to a brick-colored tenacious substance.

These symptoms continue with but slight variations for from 5 to 8 or 9 days, when the temperature suddenly falls; the pulse becomes softer and less frequent; the breathing easier; the cough loosens and the patient is a convalescent. When death occurs it is when the infectious element has reached its acme, and the heart has been overpowered.

Rutherford, of the last century, lost one in three of his cases of pneumonia; he bled largely. Laenec lost one in seven; he antimonied largely. Bennett claims to have lost only one in thirty-two by his expectant plan, but it must be remembered that this included all forms of pneumonia, and I suspect a considerable number of simple bronchitis and pleuritis. It has been the experience of medical men since Bennett's time that 75 per cent. of all cases of croupus pneumonia will recover with no treatment other than dietetic. According to the German authorities ten or fifteen of the remaining twenty-five may be saved with proper treatment.

Since we have recognized this disease as due to a specific cause, our object should be to sustain the system and not to combat the disease, and since the fatal result is due to the failure of the heart's action it should be our object to sustain that organ, and as the high fever is the principal factor in producing that failure, the first indication in the treatment is to lower the temperature. When the fever abates the force of the disease is broken.

How are we to lower the fever? The Germans do it by extracting the heat directly from the body with the cold bath. This is claimed by Juergensen to have proved wonderfully successful.

Antipyretic remedies are largely used not only for the purpose of extracting the heat, but to diminish its production. Quinine is the preferable one because it has no exhausting effect upon the heart. It is given in very large doses, from 45 to 77 grains every alternate day. Salicylic acid is employed in even larger doses for its antipyretic properties; salicylate of soda is sometimes preferred to the acid; crysophanic acid is also employed for its apyretic effects. Veratrine, by its controlling action upon the heart and (as some claim) by its strengthening action upon that organ, proves to be a valuable remedy. Aconite is used for its inhibitory action upon the heart. Stimulants, of course, are indicated in asthenic cases, since they not only spur the heart to renewed action, but directly enable it to perform more work. Opium and its preparations are valuable in relieving pain. Flannel cloths wrung out in hot water promote diaphoresis and a local anodyne action, and at the same time relieve the overcharged heart by promoting an increased peripheral circulation.

